# Understanding health professional role integration in complex adaptive systems: a multiple-case study of physician assistants in Ontario, Canada

**DOI:** 10.1186/s12913-020-05087-8

**Published:** 2020-04-29

**Authors:** Kristen E. Burrows, Julia Abelson, Patricia A. Miller, Mitchell Levine, Meredith Vanstone

**Affiliations:** 1 Department of Family Medicine, McMaster University, Hamilton, Ontario Canada; 2 Michael G. DeGroote School of Medicine, Physician Assistant Education Program, McMaster University, Hamilton, Ontario Canada; 3 Department of Health Research Methods, Evidence and Impact (HEI), McMaster University, Hamilton, Ontario Canada; 4 School of Rehabilitation Science, McMaster University, Hamilton, Ontario Canada

**Keywords:** Physician assistant, Interprofessional care, Case study research, Health policy, Qualitative research, Complex adaptive systems

## Abstract

**Background:**

To meet the complex needs of healthcare delivery, the Ministry of Health and Long Term Care (MOHLTC) introduced Physician Assistants (PAs) into the Ontario health care system in 2006 with the goal of helping to increase access to care, decrease wait times, improve continuity of care and provide a flexible addition to the healthcare workforce. The characterization of healthcare organizations as complex adaptive systems (CAS) may offer insight into the relationships and interactions that optimize and restrict successful PA integration. The aim of this study is to explore the integration of PAs across multiple case settings and to understand the role of PAs within complex adaptive systems.

**Methods:**

An exploratory, multiple-case study was used to examine PA role integration in four settings: family medicine, emergency medicine, general surgery, and inpatient medicine. Interviews were conducted with 46 healthcare providers and administrators across 13 hospitals and 6 family medicine clinics in Ontario, Canada. Analysis was conducted in three phases including an inductive thematic analysis within each of the four cases, a cross-case thematic analysis, and a broader, deductive exploration of cross-case patterns pertaining to specific complexity theory principles of interest.

**Results:**

Forty-six health care providers were interviewed across 19 different healthcare sites. Support for PA contributions across various health care settings, the importance of role awareness, supervisory relationship attributes, and role vulnerability are interconnected and dynamic. Findings represent the experiences of PAs and other healthcare providers, and demonstrate how the PAs willingness to work and ability to build relationships allows for the establishment of interprofessional, collaborative, and person-centered care. As a self-organizing agent in complex adaptive systems (i.e., health organizations), PA role exploration revealed patterns of team behavior, non-linear interconnections, open relationships, dynamic systems, and the legacy of role implementation as defined by complexity theory.

**Conclusions:**

By exploring the role of PAs across multiple sites, the complexity theory lens concurrently fosters an awareness of emerging patterns, relationships and non-linear interactions within the defined context of the Ontario healthcare system. By establishing collaborative, interprofessional care models in hospital and community settings, PAs are making a significant contribution to Ontario healthcare settings.

## Background

Physician Assistants (PAs) are advanced clinical practitioners trained in the medical model to extend physician services, and are currently employed in a wide variety of healthcare settings across a number of countries, including the United States, United Kingdom, Australia, and the Netherlands [1–3]. The role of PAs may offer solutions to the inherent tensions between service demands, training requirements and budgetary restraints, compensate for cyclical health workforce shortages, provide a flexible addition to the healthcare workforce, provide team continuity, and improve patient experiences [4–6]. Hoping to achieve similar results in Ontario (Canada), the Ontario Ministry of Health and Long Term Care introduced PAs in 2006 as a potential health human resource innovation to improve access to care, reduce wait times, and support the complex needs of healthcare delivery in Ontario [7–9].

Despite the growing interest in PA education, integration and uptake of employment across the province, few studies have explored the PA role in Ontario, especially from the perspective of PAs, other healthcare providers (e.g., physicians, nurses, residents), and administrators. Limitations to PA research are attributed to a lack of comparator groups, poor study setting descriptions, and the consideration of evidence from the United States where context (i.e., healthcare funding) is often different [10]. Compounding the dearth of research evidence are a number of barriers that limit PA role sustainability, including lack of health professional regulation, unstable funding sources, and resistance from other health care providers.

Canada currently offers PA training through one military training program (Canadian Armed Forces, students are military personnel) and three civilian training programs (McMaster University, Hamilton, Ontario; Consortium of PA Education, University of Toronto, Ontario; University of Manitoba, Winnipeg, Manitoba) [7–9]. Each of these programs are two years in duration, and successful graduates are then eligible to challenge the National Certification Exam, which provides the CCPA designation (Canadian Certified Physician Assistant). Civilian PAs (i.e., non-military) are currently employed in the Canadian provinces of Ontario, Manitoba, Nova Scotia, Alberta and New Brunswick, but are only recognized as either a registered or regulated health profession in Manitboba, New Brunswick and Nova Scotia. Alberta is currently awaiting legislative changes regarding PA regulation.

PAs were introduced as one potential health care innovation to induce change at a systems level and their ongoing integration requires careful documentation and analysis. Understanding the relational aspects of care delivery is critical for innovation success, and being deliberate about interdependencies and their role will lead to improved interventions, especially in the context of policy change that promotes effective coordination and communication among health care providers [11]. Analysis of the diffusion of this health systems innovation is constrained by a lack of information on which processes enable and sustain implementation in health service delivery and organizations, the context of how the innovation (i.e., PA role integration) is situated in particular settings, and whether these processes can be enhanced and replicated [12].

We approach the examination of the role of PAs in the healthcare system through the theoretical lens of complexity theory, and consider the PA to be one agent in the complex adaptive system (CAS) of healthcare. Health care systems are nonlinear, dynamic and unpredictable and are comprised of a network of components (e.g., hospitals, clinics, families, patients) that interact nonlinearly on different levels (e.g., patient, medical center, government) [13]. Complexity theory, or CAS, suggests that the key to understanding the healthcare system is examining the patterns of relationships and interactions among the system’s agents [14], which lends itself to exploring the role of PAs as new members of Ontario healthcare teams.

The aim of this study is to explore PA role integration in the Ontario healthcare system through an in-depth analysis of setting and role descriptions, described outcomes, and healthcare provider perceptions. This investigation is organized around the research question: What factors influence successful PA integration in Ontario, Canada? By additionally examining the role of PAs as agents in the health care system through a complexity theory lens, this study will provide additional insight on the relationships and interconnections that frame PA role integration and contribute to broader health services research.

## Methods

### Study design

In order to examine PA integration in Ontario, an exploratory multiple case study approach was chosen. Case study methodology allows the researcher to focus on the selection of rich cases that provide context to the research questions or phenomenon of interest. Exploring multiple cases allows the researcher to understand the differences and the similarities between cases [15, 16], and to analyse the data within and across sites [15]. The evidence generated from multiple case studies allows for a wider discovery of theoretical evolution and research questions, thus creating a more convincing theory [16]. This study design and methodology was based on the rationale that the study of health professional role integration is complex, context dependent, and involves social processes [17]. This method also allows for the in depth exploration of setting specific contextual factors, the identification of consistent factors by analysing across cases, and for knowledge to be developed about how and why some events and situations affect others [18].

**Fig. 1:**
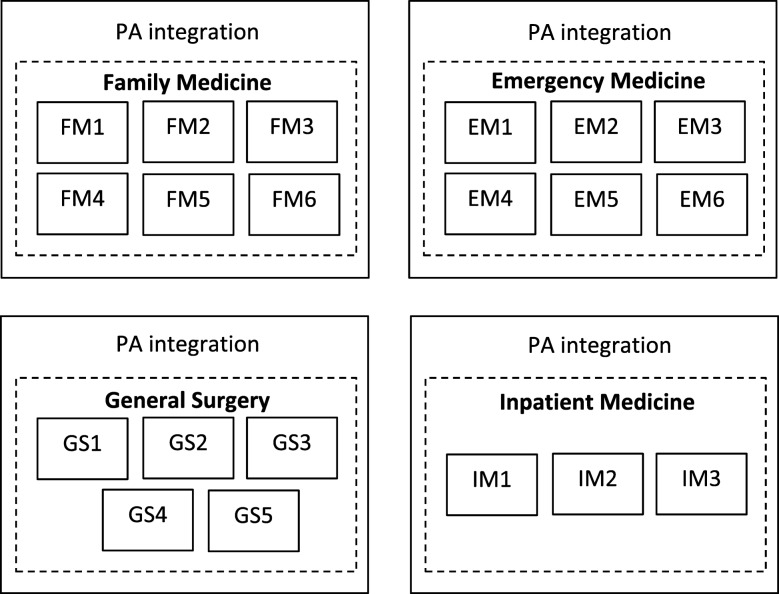
Schematic of Multiple-case Design with Embedded Subunits of Analysis

Four purposefully selected health care settings that employed PAs (i.e., family medicine, emergency medicine, general surgery, and inpatient medicine which included the cardiac intensive care unit or internal medicine) were chosen as the cases (Fig. 1). PAs were recruited via convenience sampling from a range of different sites within each of the four healthcare settings and were selected based on meeting study criteria. Within each case/setting, individual practice sites were selected as embedded subunits of analysis. This multiple-case study is bounded by the Province of Ontario, and the phenomenon of interest is the successful integration of PAs into Ontario health care settings. “Successful” settings were defined as sites where the PA had been employed for a minimum of two years, and were either permanent full time employees, or were eligible for ongoing contract work.

### Data collection

Data sources included key informant interviews, site-specific documents, and government communications relating to the PA role. Key informants (i.e., participants) were recruited through the Canadian Association of Physician Assistants (CAPA) email distribution list, and postings on the Ontario PA Facebook group. PA participants who met the inclusion criteria were asked to recruit other members of their respective healthcare teams with assistance from the research team. Inclusion criteria specified that the PA had to be employed in one of the four settings of interest for a minimum of 2 years to participate.

Semi-structured interview guides (example provided in Additional file 1) were developed for PAs, residents/learners, physicians, administrators/managers and other health care providers who worked directly with the individual PA at their respective site. The interview guides were structured around the identified theoretical propositions as informed by evidence, grey literature, and personal experience:

1. The generalist medical training of PAs has the potential to impact role definition in a dynamic healthcare system;
2. Barriers and facilitators to PA integration are interconnected and relationship dependent;
3. Physician knowledge and experience with the PA role impacts role integration success;

The interview guide queried components relating to the PA role, how the role has been accepted, and any facilitators or challenges that had arisen since the PA role was introduced within the four settings. Participants were given the option of phone or in-person interviews, which typically lasted 30–45 min. Each interview was recorded and transcribed verbatim. Interviews were conducted by the first author (KB), the local principal investigator (MV), or a research assistant depending on participant scheduling, researcher availability, and to mitigate any potential conflicts of interest (e.g., when the research participant was known to a member of the research team).

Site-specific documents relevant to the role of the PA (e.g., medical directives, job descriptions/ postings, media publications, and organizational websites relating to the PA role) were collected from participants and publicly available sources in order to provide context around PA role integration at each site. Existing and archived policy documents from various provincial stakeholders were also reviewed in order to understand the context of provincial stakeholder initiatives. These included communications and position statements from the Ontario Medical Association (OMA), Ontario Hospital Association (OHA), College of Family Physicians of Canada (CFPC) and HealthForceOntario (HFO) [19–21]. This concurrent document analysis helps support the narrative describing the PA role, and situates the interview data in a broader policy and organizational (i.e., hospital setting) context.

### Data analysis

Interviews were transcribed verbatim and data analysis was managed through N-Vivo version 12. Data were analyzed in three phases:

- *Phase I* consisted of a case description (explanation building) and an inductive thematic analysis for each of the four case settings (family medicine, emergency medicine, general surgery, and hospital inpatient medicine).
- *Phase II* involved a thematic cross-case analysis in order to identify crosscutting themes to explore the similarities and outliers across the four case settings. Outliers were defined as unique perspectives or case exceptions that deviated from the central themes, or were discordant to other case/setting characterizations [22].
- *Phase III* consisted of a deductive exploration of the identified cross-case patterns and themes pertaining to complexity theory, especially around CAS principles related to relationships, interconnections and uncertainty. Interpretation of the data entailed identifying key concepts that explain relationships between the themes and theoretical assumptions, in addition to highlighting messages that are relevant to policy makers.

The primary investigator (KB) coded each transcript. A random sample of interview transcripts were coded by a second reviewer (either a research assistant or PM) in order to ensure data congruency. Emerging themes, patterns, and case outliers were discussed amongst the full research team, which included a physician assistant (KB), physiotherapist (PM), physician (ML) and two non-clinician health systems and policy researchers (JA, MV).

**Fig. 2:**
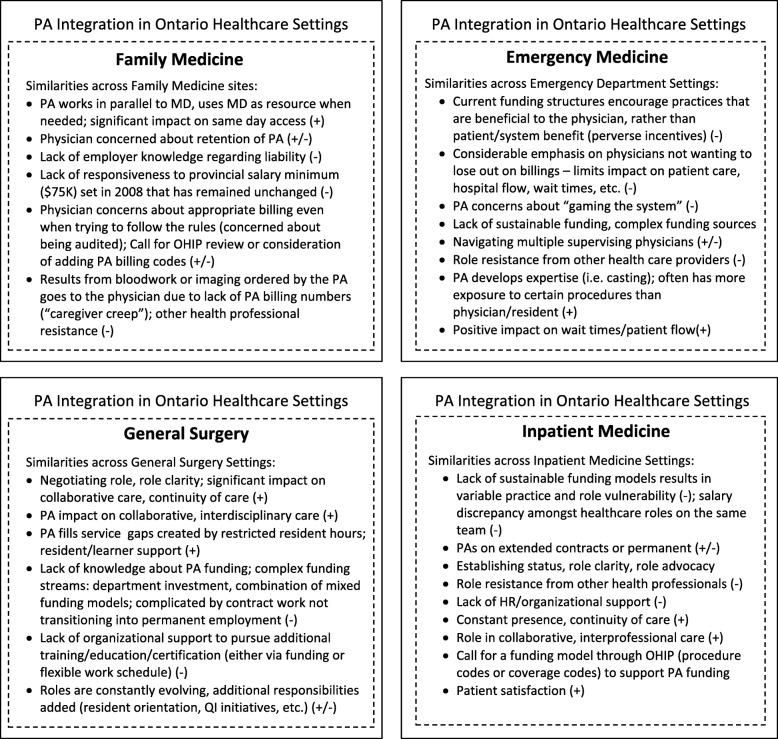
Summary of Within-Case Analysis (overarching themes within each case)

**Fig. 3:**
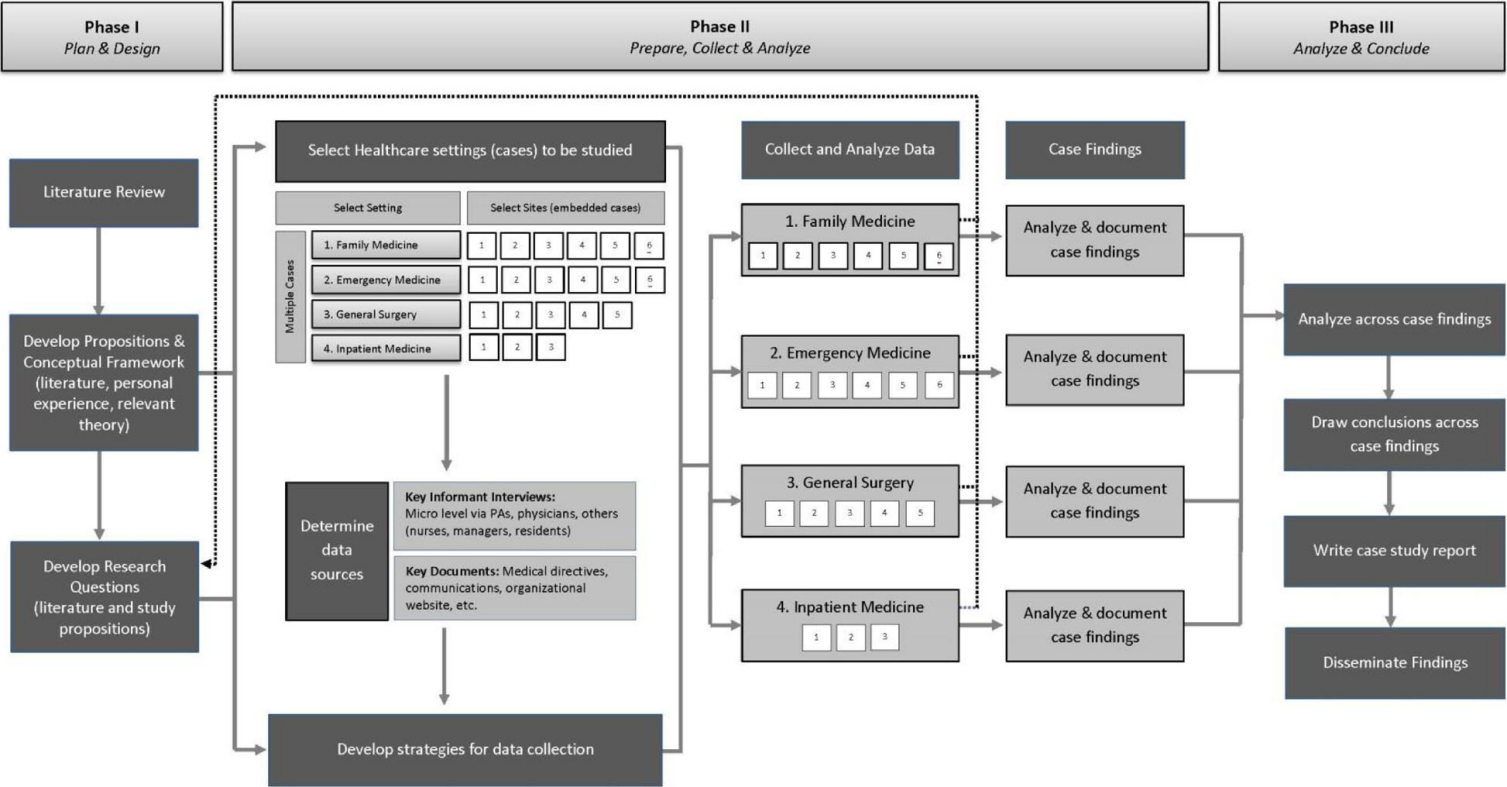
Overview of Multiple Case Study Design & Approach. Adapted from Yin [23], Sangster-Gormley [24], and Whitmore [25]

The multiple-case analysis started with the development of a description of each case setting [15] (Table 1). The process of identifying the factors/themes that fit each case (family medicine, emergency medicine, general surgery and other inpatient hospital settings) was an iterative process, cycling back and forth between the emerging themes and case data. Factors and processes were only included if they were supported by the data (as documented in the chain of evidence) and if they related to the initial study propositions and research aim. Themes were generated for each case setting and were then reviewed in the context of the other settings to determine cross-case similarities and to determine outliers. Given the extensive volume of data generated from each case, details on the development of each theme are not provided. A summary of the each case analysis, including details on the embedded case sites, is presented in Fig. 2, and an overview of the full research protocol and approach is demonstrated in Fig. 3.

**Table 1:** Characteristics of case settings and embedded sites

Multiple Case Study Settings				
Sites:	Case 1: Family Medicine (FM)	Case 2: Emergency Medicine (EM)	Case 3: General Surgery (GS)	Case 4: Inpatient Medicine (IM)
Embedded sites	6 Family Practices; Mix of urban (5 sites) and rural (1 site); mix of academic (4 sites) and non-academic practices (2 sites).	6 Emergency Departments; Mix of urban (4 sites) and rural sites (2 sites), mix of academic (5 sites) and non-academic hospitals (1 site)	5 Hospitals; mix of rural/non-academic (1 site), and urban/academic hospitals (4 sites)	3 Hospitals; All urban sites, all academic hospitals (3 sites)
Interview Data	Semi-structured Interviews (16); 7 PAs, 8 Physicians, 1 Clinic Manager	Semi-structured Interviews (13); 7 PAs, 5 Physicians, 1 RPN	Semi-structured Interviews (12); 5 PAs, 3 Surgeons, 2 Surgical Residents, 2 IP Directors (MD, RPN)	Semi-structured Interviews (5); 4 PAs, 1 Physician
Document Data	Documents: medical directives, integration tool kits, HFO website/ communications, Patient’s First Document, 2011 College of Family Physicians of Canada position statement on PAs	Documents: medical directives, job postings, HFO website/ communications, organizational websites, media/news	Documents: medical directives, job postings, HFO website/ communications, organizational websites, media/news; OHA position statement on PAs; surgery department handbooks	Documents: medical directives, HFO website/ communications, organizational websites; OHA position statement on PAs
Description of PA role	Certified Canadian (civilian and military) and United States trained PAs with 2–9 year of family medicine experience at the time of data collection.	PAs were all Canadian Certified (CCPA) and had been practicing in emergency medicine for 4–9 years at the time of data collection.	PAs were all Canadian Certified (CCPA) and had been practicing in general surgery for 2.5–5.5 years at the time of data collection.	PAs were all Canadian Certified (CCPA) and had been practicing at their hospital site for 2–5.5 years at the time of data collection.
PA-MD supervisor relationship	PA/MD work collaboratively, often in parallel. Relationships are longitudinal. PA usually supervised by 1 primary physician.	PA/MD work in same general department, but might be assigned to different areas to different patient cohorts (i.e. triage or assigned different CTAS level patients). PA works with multiple supervising physicians.	PA/surgeon work in same department, but surgeon often in OR. PA present on the ward and for consults within the ED and hospital. PA works with multiple rotating supervising physicians. PA is continuously available.	PA/MD work in same department, but may divide patients between team or may be assigned different tasks. PA works with multiple supervising physicians, so becomes centre of continuity.

### Methodological standards: Validity & Reliability

Pattern matching, explanation building and replication logic were used to establish overarching associations across each of the four cases [15]. Reliability was supported through adhering to a case study protocol. The case study protocol included case selection criteria, interview guides for each member of the healthcare team, and a database of collected documents. Validity was reinforced by using multiple sources of evidence (e.g., medical directives, new media articles, and organizational websites), establishing a clear chain of evidence, and using multiple researchers to code data derived from the interview transcripts, and to address rival explanations. Each of these case study strategies also helped support the trustworthiness of the research study by establishing credibility (through triangulation and peer debriefing), transferability (descriptive explanation building), and confirmability (reflexivity and documenting the chain of evidence) [26].

### Data sufficiency

After case selection has occurred, the determination of data sufficiency relates to a rich description of the selected case(s). Strategies such as using multiple sources of data (interviews, documents, archival records, etc.), data triangulation, and maintaining a chain of evidence and audit trail help support data sufficiency [15, 27, 28]. This multiple case study was data rich, contextual, and involved multiple sources of evidence that generated a substantial volume of qualitative data. The number of data sources was appropriate to the complexity of the study topic and the depth of data collected from each setting [15].

### Ethical considerations

This study was reviewed and approved by the Hamilton Integrated Research Ethics Board (HiREB), as documented in protocol #2270. Each participant in this study provided verbal informed consent prior to their interview.

## Results

Forty-six health care providers and administrators were interviewed across 19 different healthcare sites (hospitals = 13, community clinics = 6), including 24 physician assistants, 17 physicians, 2 medical residents, 2 registered nurses, and 1 family health team administrator.

Although there are some variations between practice settings, such as the time and nature of physician collaboration and number of supervising physicians, there were numerous similarities identified in the cross-case analysis. Four interconnected themes emerged from this multiple-case analysis: PA role contribution to Ontario healthcare settings; developing role awareness and role clarity; supervisory relationship dynamics; and variability in funding and remuneration (Table 2). In addition, a number of outliers are presented within the context of the cross-case analysis. These outliers represent experiences, outcomes or exceptions that deviated from the main emerging themes.

**Table 2:** Summary table of themes from cross-case analysis

**CROSS-CASE THEMES AND PATTERNS**	
**1. Contribution to Ontario Health Care Settings**	
• Idea of a versatile, flexible, responsive, accessible health care provider that models collaborative, interprofessional care (stemming from foundation of core professional competencies)(+)	
• Focus on person-centered care – nature of the role allows for time, education and advocacy on behalf of patients(+)	
• Patient navigator – navigates community resources, hospital resources, other services, etc. (+)	
• Increase access to care – allows for increased patient volume, decreased wait times, same day appointments, faster consults, timely discharges (+); Fill gaps/bridging gaps in the health care system(+)	
• Significant impact on improving continuity of care (+)	
• Leadership & support – mentorship of learners, support residents, interest in research opportunities, quality improvement initiatives, other committee work, etc. (+/−)	
• Cost of the role; organizational role (+/−)	
**2. Developing Role Awareness & Role Clarity**	
• Presence of a PA advocate or champion (+)	
• Challenge of working as an unregulated health care provider (lack of regulation); understanding of delegation, controlled acts and use of medical directives (+/−); Knowing when to seek help, knowing what you don’t know(+)	
• Trajectory of role development: how PA or role was initially introduced; PA transition to practice; PA establishing role and functioning effectively (learning curve) (+/−)	
• Access to resources/supports (administrative, physical space, CPD funding/time) (+/−)	
• Navigating role and work environments amongst residents (especially in academic centres); how PA role is introduced to a learner, i.e. medical students, residents, etc. (+/−)	
• Heavy reliance on PA to deliver services; role evolution (+/−)	
• Organizational support; level of autonomy; influence of patient satisfaction (+/−)	
• Incentives (financial, time, support) to provide administrative, teaching or mentorship to medical students, residents, or PA learners (−)	
• Other healthcare professions not understanding role, not accepting orders, interprofessional relationships(−)	
• Concept of “caregiver creep”: PAs don’t have an individual or MD-associated billing number, blood results ordered by the PA go back to the physician provider rather than the PA, even if the PA has been regularly seeing patient. Leaves providers feeling as though they have lost their role as care provider (−)	
• Lack of evaluation processes (performance, patient flow, productivity)(−)	
**3. Supervisory Relationship Dynamics**	
• Nature of supervisory relationship allows PA to learn from a variety of practitioners – PA is exposed to variety of practice styles, personalities, bed-side manner, medical expertise, other consulting services, etc. (PA can adapt their own practice style by observing others, determine what works best for their own setting/clinical environment – echoed across settings where multiple supervising physicians are part of daily practice)(+)	
• Role of trust and mutual respect, defining entrustment, presence of PA frees up physician for other patients/cases (+)	
• Mutual support/resource: PA develops skill set that extends Physician services, or PA becomes the procedure or content expert due to frequency of exposure and clinical experience (+)	
• Mutual learning curves: PA orientation to clinical setting, procedures, physician preferences; Physician orientation to working with a PA (+/−); Physician experience, PA background (training, specialty interest) (+/−)	
• Feeling alone, lack of supervisory oversight (−)	
• Physician knowledge of oversight and liability (−)	
**4. System Variability and Sustainability**	
• Potential disconnect being the physician supervisor +/− employer that has implications on sustainability of role, vulnerability of PA role, and PAs ability to negotiate for equal/more pay (+/−)	
• Navigating an unknown future; need to appropriately shift resources (+/−)	
• Variable remuneration for additional responsibilities (i.e. teaching, mentorship, QI initiatives, research)(+/−)	
• Inconsistent funding models, funding sources, salaries, benefit packages, and hourly rates; Lack of clarity around funding sources, streams, and opportunities (−)	
• Poor responsiveness to cost of living standards, stagnant salaries(−)	
• Concerns about “gaming the system”; double billing (−)	
• Lack of PA specific management or advocacy for contract negotiations and role sustainability (−)	

Impact on Role Optimization (+ or -)

**(−)** Factor or process negatively impacts role optimization (is a challenge or barrier)

**(+)** Factor or process positively impacts role optimization (facilitates or supports role optimization)

**(+/−)** Factor is neutral, or in some circumstances, it can act as a barrier; in other settings, it is a facilitator

### Role contribution to Ontario health care settings

The PA role provides a versatile, flexible, and accessible health care provider who models collaborative, interprofessional care in complex settings. Favorable contributions of the PA include increasing patient access to care, fostering person-centered care, improving continuity and filling gaps in the health care system.

In addition, PAs take on a large amount of administrative work, such as patient care documentation, discharge summaries, dictations, consult requests, and resident/learner orientation, which helps improve patient flow.

*“The success has been that they’re part of … a team that has taken a program with 1,200 cases and gone to 1,800 cases, with the same number of beds, right. They’ve become a significant part of our improvement and operations … We had all kinds of budget problems with … physician coverage, so they were also an economic success … a tangible reduction in costs for human resources during the day.” [MD, IM]*

One unique contribution of PAs is the flexibility and adaptability of their skill set. Across all settings, physicians and PAs provided examples of where being consistently present in their setting or working with particular patients allowed the PA to become a procedure or content expert due to frequency of exposure and clinical experience, or develop a skill set that extends physician services:

*“I think because I’m there every day and the doctors rotate, I’ve actually probably performed more of those procedures than most of the* [physicians] *I work with” [PA, EM]*

This expertise is reflected in physician feedback that described how other consulting services (e.g. orthopedic surgeons) started to prefer getting consults from the PA because of the PA’s understanding of the precise information that the consulting service requires:

*“They’re so specialized and they see all of those cases, so* [they’ve learned] *exactly what each specialist wanted,* [they’ve learned] *how they wanted them cast, they really paid attention to these details that 30* [emergency doctors], *who don’t get the same volume and maybe aren’t interested in the same way … so* [there’s] *really a great deal of satisfaction among orthopedic specialists who take referrals from the PAs.” [MD, EM]*

One identified case outlier involves the interplay between increased patient volume and other setting-specific considerations. In settings such as general surgery, increased patient volume in the emergency department (e.g., patients waiting for a surgical consult) or an overloaded ward (e.g., arising when surgical beds are filled to capacity) puts a strain on staff because of the number of consults to be seen, pending discharges, and additional families to update. Faster surgery turnarounds facilitated by PAs may mean a higher need for recovery and ward beds, which were not always available. In contrast, physicians at Family Medicine sites were enthusiastic about the ability to handle increased patient volume because this meant increased access to care for patients and increased remuneration for physicians.

### Developing role awareness and role clarity

The importance of role awareness and establishment of role clarity was echoed by all participants across the four settings. PA participants described both benefits and challenges associated with being an unregulated health care provider:

*“Being unregulated is also a big thing, because now … unions in the hospitals that have a strong union presence, being unregulated does raise a lot of questions, especially when there are budget cuts … and then they start bringing in different levels of providers that aren’t regulated. It creates a bit of tension.” [PA, GS]*

In addition to challenges around lack of regulation, participants also reflected on the complexity of navigating delegation, controlled acts and variable uptake of medical directives. As others in the network of PA care (e.g., patients, health care providers, and administrators) became more aware of the PA role, role clarity is gradually established. Participants acknowledged the importance of organizational support; both for when the PA role is first introduced, and as it pertains to successful integration and role evolution.

Ultimately each case setting is now heavily reliant on the PA to deliver services, including day-to-day patient care, quality assurance initiatives, other administrative roles (i.e., lead PA), and resident or learner orientation and teaching. Navigating role and work environments with medical learners and residents can be challenging as the potential exists for challenges around role clarity and overlap. However, these can be ameliorated by an appropriate orientation of learners to the team players and roles within a site that employs a PA. Physician perception is an important driver of this role clarity:

*“A resident is there to learn; their primary responsibility is towards their education. PAs are also learning and everything we invest in them we get back. But at the same time, the PAs have a bigger responsibility to manage flow, so they are more efficient generally than residents are, and they are always there … they’re not having to relearn the process” [MD, EM]*

Unfortunately organizational and physician support can be undermined by other healthcare professions who may not understand the role, not accept orders written by the PA, or actively demonstrate resistance to role integration: *“I know other pharmacies have a hard time understanding the role of PA and reject some prescriptions” [PA, FM],* thus decreasing service delivery and efficiencies. In all settings, the PA’s enthusiasm, self-organization and role awareness enables the PA to either change perceptions or find strategies to maximize efficiencies.

With respect to case outliers, it was clear that the PA role is most easily defined by all team members in Family Medicine settings. This is likely influenced by the longitudinal nature of the PA-MD-patient relationship, and the parallel practice of the PA and MD. In emergency settings, any impact of continuity of care is limited to the PA-MD shift schedule and role definition is less controversial due to the close proximity of the work environment. PAs and MDs are seeing patients, interacting with nursing staff, and updating families in close geographical proximity, and opportunities to discuss a patient are more available. In general surgery and other inpatient settings, role clarity is more complicated due to turnover of residents, patients, surgeons/staff physicians in the midst of new consults, discharges and larger interprofessional healthcare teams.

### Supervisory relationship dynamics

A key characteristic of the PA-physician relationship is trust, and the development of trust is influenced by the physician’s understanding that the PA knows when to seek help. The physician must trust the PA to seek help, and the PA needs to feel confident that the supervising physician is readily available for consultation when required. Failure to seek help or support the PA negatively impacts the relationship dynamics.

*“If it’s a new doc, or I’m unfamiliar. Or if they’re a new hire and haven’t worked with a PA, they’re going to want to review most patients with us. But again it depends. It’ll also depend on my comfort level with a patient. If* [the patient] *is presenting* [with something] *I’m really not familiar with, or I feel that the patient is a lot more sick than I’m comfortable dealing with, then absolutely I’ll bring in my* [physician] *much sooner than otherwise.” [PA, EM]*

The nature of the supervisory relationship allows PAs to learn from a variety of practitioners. There is considerable setting-dependent variability in the number of supervising physicians that work with a PA (ranged from 1 to 18). PAs are therefore exposed to a variety of practice styles, personalities, bed-side manners, medical expertise and other consulting services. PAs can then adapt their own practice style by observing others and determining patterns that work best within their own setting and clinical environment: *“I appreciate and enjoy* [different practice styles] *and I think it’s nice that it allows me to be able to see all kinds of styles and create my own” [PA, FM]*

Working with multiple supervising physicians also requires the PA to constantly adapt their own practice as *“everyone has a slightly different clinical approach” [PA, FM]* that requires the PA to *“deal with multiple personalities” [PA, GS].* Negative interactions occurred when the PA felt alone or felt as though they lacked supervisory oversight: *“I was somewhat left to my own devices at times when I feel like help might be needed, and help’s not always readily available when the rest of the team is in the operating room” [PA, GS].* In addition, variable physician knowledge regarding liability and supervision was identified across each case setting.

Family medicine sites had a significantly reduced number of supervising physicians, compared to the other cases/settings. The family medicine PA-MD team are more likely to work in parallel, with both seeing their own patients and reviewing patient information together only when necessary. In settings with multiple supervising physicians, the PA must also adapt to a variety of practice styles and preferences, which can be a benefit (i.e., can adapt their own practice style) or a hindrance (i.e., there can be varying levels of autonomy that require the PA to constantly adjust their approach to satisfy the supervising physician).

### Impact of system variability on funding and remuneration

Across all four settings, funding was consistently identified as a challenge. PAs stated, *“I’m not satisfied* [with remuneration] *because we are still at the same rate as actually, a little less, than when I was hired over 5 years ago, so that’s very frustrating” [PA, EM],* or that *“There has been very little increase. I do have job security which is nice, but there are absolutely no benefits, no increase in vacation [time] … there’s been nothing, so that’s very frustrating” [PA, FM].* In addition to dissatisfaction with their salary, cross-case analysis revealed very little employer/organizational responsiveness to consideration of incremental cost of living increases. Most PAs reported that their salaries have remained unchanged since the PA role was introduced to Ontario in 2006.

Funding comes from multiple sources, including global hospital budgets, departments, pay-for-performance, other allocated funding sources (i.e., Family Health Team allied health funds) or directly from physicians. The challenge of these variable sources is the dependency on intermittent, short stream funding and its impact on role sustainability. One Emergency Physician described the precariousness of funding PAs based on their contribution to meeting a pay-for-performance incentive to reduce wait times:

*“The danger is that if our* [department] *performance went down, then we would no longer be able to afford* [our PAs] *or if the province stopped the program, we would no longer be able to afford them. So our PAs live in fear every year, because they do not have stability in their jobs. They do not have contracts; they do not have job safety.” [MD, EM]*

Physicians and PAs across all settings called for a re-examination of funding and regulatory status:

*“My wish would be that there’s some funding model that comes up through OHIP (Ontario Health Insurance Plan) that would pay for them; procedure codes or coverage codes or something so there’s some funding available for* [PAs].” *[MD, IM]*

“*… Hospitals are constantly having to cut the budget; and that’s kind of what we’ve been running into lately is, more and more were being kind of asked to prove, not so much prove, we’ve proven our work; but we’ve been essentially told that, we love you guys, but we can’t necessarily fund you forever” [PA, EM]*

One noteworthy case outlier relates to evidence of inappropriate billing practices and perverse incentives in select Emergency Departments. The organization was covering the salaries for the PAs in the emergency department, while the physicians were personally billing for the services offered by the PAs: *“they cost nothing for us to have them, they generate income for doctors” [MD, EM],* or “*gaming the system*” through physician or departmental use of the PA to earn incentives. For example, in multiple cases the Emergency Department arranged PA workflow to assess patients quickly, thereby maximizing their chances of meeting a pay-for-performance target and receiving a financial bonus for reducing wait times. The downside is that patients often waited longer to then be cleared by the physician, demonstrating multiple inefficiencies and the opposite intent of the incentive:

*“Basically the whole reason you were sitting there is so you could write up a note to put a time on it … the patient came at 8 to triage, you wrote a note, showed the [physician] at 8:05, and in the records … the patient was seen in 5 minutes. But they weren’t seen in 5 minutes … now they’d go to the other waiting room and wait 2 hours to see the doctor. So their real wait time was 2 hours, but on paper it was 5 minutes, and to the government looks really good, and so then they can give the hospital more money.” [PA, EM]*

In general, billing and funding issues are more complex in hospital settings as there is often no single clear funding source; conversely, liability insurance is less of an issue in hospital settings if the PA is a hospital employee and thus covered under organizational insurance.

## Discussion

The multiple case study generated cross-case themes that helped identify the various barriers, facilitators and systemic factors that impact PA role integration in family medicine, emergency medicine, surgery and other inpatient settings. Results from the cross-case analysis establish a foundation for understanding how the PA role contributes to Ontario health care settings, the importance of developing role awareness and clarity, the dynamic supervisory interface between physicians and PAs, and the impact of system factors on role sustainability. As described in other literature, individual PAs roles are described as mouldable, which allows the PA to work across silos within the organization in order to fill the needs of their setting. Working across these barriers enables PAs to address care delivery gaps, provide continuity, increase collaborative care, enhance communication, aid patient flow, enhance care during transitions, and free up physician time for other patients or activities [4, 29, 30]. Most importantly, PAs contribute to a relationship infrastructure that enables effective communication between patients and their health care team.

The cross-case analysis revealed that factors such as funding, perverse incentives, understanding of liability, lack of regulation and role clarity are often messy and unclear across multiple settings (Table 2). These discrepancies limit long-term relationships (between the PA and employer), sustainability, role success, and the optimization of efficiencies. Understanding differences in the way that role uncertainty manifests in different clinical settings can lead to an improved understanding of the types of improvement efforts (either adaptations or enhancements) that may be more effective [11].

The characterization of PAs as one of many self-organizing agents in complex adaptive systems (e.g., health organizations) allows for barriers and facilitators of PA integration to be considered in the context of a complex network of stakeholders, interactions, events and collaborations. Across each of the four case settings, PA role integration is non-linear, dynamic, and influenced by cross-case factors. The PA role, by nature of its role diversity, flexibility, and focus on patient-centered care, embodies a dynamic approach to health care delivery that facilitates interactions between other health care providers, medical learners, patients and families. However, as one agent in a large health care organization, the success of the PA role is influenced by role uncertainty, complex funding streams, relationships (both negative and positive), and distributed control as experienced by study participants.

Reflecting on CAS theory and its relevance to this study, the results highlight the operational differences and variations in how the PA role is funded, employer or physician knowledge of funding sources, knowledge of appropriate billing practices, and role descriptions. This is reflected in the observation that the same policy objective (i.e., MOHLTC introduction of PAs) can lead to multiple local configurations and interactions, as demonstrated across cases. Some CAS studies suggest that this variability may allow for a more robust health care system that can adapt and self-organize [31], and where intrinsic properties can be exploited to guide healthcare in a more favorable direction [13]. The variation identified in the cross-case analysis demonstrates multiple different configurations of the PA role that have adapted and self-organize to best serve the setting in which the PA is employed.

The application of a complex system theory approach as it pertains to the health human resource innovation of PA integration in Ontario is novel. Although complexity theory has not been explicitly applied to the PA profession, other research supports the use of CAS to explain why interdisciplinary teams are successful in the provision of services when cases are complex [32], and to explain how patterns of interactions between health team members define team behaviour [33]. Complexity theory is well suited to explore PA role integration given the complexity of healthcare settings including multiple stakeholders, multifaceted issues, uncertainty, diverse agendas, and interconnected relationships.

Exploring PAs as one agent in a complex adaptive system was helpful for examining the iterations, complexity, emerging patterns and interrelationships that support this flexible and adaptable addition to the Ontario health care system. Complexity science provides insights that could not have been reached when only using the traditional explanatory model based on scientific positivism that describes the linear cause-effect relationship between two isolated events [33, 34]. Furthermore, CAS was helpful in offering potential solutions and routes for ongoing development of PA integration. By recognizing that the success of PA role integration is largely contingent on relationships and interconnections, removing structural boundaries between professionals, aligning their goals, enabling adaptation and experimentation, and establishing simple rules to minimize expenditures [13], will help optimize the PA role and its sustainability.

### Strengths and limitations

The multiple-case study approach allowed for the exploration of relationship patterns, interactions, and processes, and was essential to understanding the successes and challenges of PA role integration within complex adaptive systems. By using a case study approach with attention to relationship patterns, study results are richer and may afford more opportunities for potential interventions [14, 15]. As more than one case was studied, multiple successful patterns of PA role integration were identified, providing examples of self-organization and similarities in a variety of healthcare settings.

One limitation regarding use of complexity theory is variability in the application of core CAS principles which may lead to conceptual confusion [35]. This limitation did indeed lead to challenges in the operationalization of complexity theory within this research study, as multiple resources reference slightly different principles or features [33, 35–40]. However, the methodological approach of iterative coding and thematic analysis within and between cases, in addition to application of complexity theory, allowed for the identification and refinement of the CAS features most relevant to participant experiences.

Additionally, this research focused exclusively on settings where PA role integration was deemed a success. Case selection targeted PAs that had been employed for at least 2 years, which means the study design did not capture settings where the PA role had been terminated due to system or funding issues. The recruitment of successful, well-functioning PA role integration was partly balanced by participants disclosing negative experiences in previous practices or during their initial implementation. Finally, this study had very limited participation from other non-physician health care providers and did not directly elicit patient satisfaction.

### Recommendations for practice, policy and research

Multiple studies echo the importance of determining the appropriate level of regulation and funding support to fully realize the utility of PAs and to optimize their integration [4, 5]. In addition, the lack of policy around reimbursement and incentives means that existing payment incentive mechanisms and the legacies of past policies are driving various stakeholders to pursue divergent interests (i.e., physician interests – save time, optimizing their income/reimbursement; increase patient access to care vs. PA interests – to be valued and acknowledged, improve patient experience, negotiate level of autonomy, etc.). The current policy vacuum sets up a system that has the potential for PAs to be taken advantage of as they constantly “need to prove their value” in order to have achieve sustainable employment. Health professional roles are shaped by professional regulations, organizational routines, and interpersonal relations, and therefore give rise to unforeseen events [39]. In addition, misalignment at any point in the system to accurately predict how a policy will be implemented, can lead to precarious success or policy (i.e., role integration) failure [41].

To help guide future policies and avoid unanticipated consequences, policy makers should approach health care as a complex system [13] and proactively think about the likely effects and full range of actors and stakeholders [42]. Being more deliberate about exploring patterns and relationships around role integration will lead to improved interventions, particularly around reimbursement models and policy changes that promote effective coordination and communication amongst providers [11]. Establishing a regulatory body, re-examining current government physician reimbursement models, competitive salary structures, and minimizing perverse incentives will all contribute to PA role optimization. From a practice perspective, PAs and interested employers should continue to voice their successes and challenges. PA enthusiasm, flexibility, and adaptability should be nurtured and supported in healthcare settings, especially where high-physician turnover, patient volume, and teaching requirements challenge collaborative and interprofessional care opportunities.

## Conclusions

This study explored and identified key factors that support or restrict the optimization of PA role integration across multiple case settings in Ontario. The exploration of PA contributions across various health care settings, the importance of role awareness, supervisory relationship attributes, and role vulnerability (in relation to sustainability and funding) are interconnected and dynamic in surgical, inpatient, emergency department and family medicine settings. These findings represent the experiences and perceptions of physician assistants, physicians, and other healthcare providers (i.e., nursing, administrators) and demonstrate how the PAs willingness to work and ability to define their roles within existing structural frameworks allows for the establishment of interprofessional collaborative person-centered care. The individual determination of practitioners to make it work was crucial for role success in light of numerous challenges posed by system structures at policy and practice levels.

The exploratory design of case study research allowed for the identification of similarities and differences across a variety of Ontario healthcare settings that employ PAs. Complexity theory was particularly helpful for studying the PA role within dynamic relationships, adaptable interactions, and unpredictable health care settings. PAs are playing a vital role in the delivery and support of healthcare within a multitude of settings as adaptable and collaborative team members focused on person-centered care. As the PA profession continues to expand into new jurisdictions, findings from this study help fill existing knowledge and practice gaps regarding the role of PAs. Documenting the central role of PAs will continue to inform the design and dissemination of research in order to optimize health care system efficiencies though PA integration.

## Supplementary information

**Additional file 1.** Sample Interview Guide.

## Data Availability

The interview data generated and analyzed during the current study are not publicly available to protect patient confidentiality, but are available from the corresponding author on reasonable request. Supporting data (government documents, medical directives, media publications) are publically available through the following websites: Canadian Association of Physician Assistants (https://capa-acam.ca/); HealthForceOntario (http://www.healthforceontario.ca/en/Home/Health_Providers/Physician_Assistants); Nexis Uni® (https://advance.lexis.com)

## Abbreviations

CAPA: Canadian Association of Physician Assistants
CAS: Complex Adaptive Systems
CCPA: Canadian Certified Physician Assistant
CFPC: College of Family Physicians of Canada
EM: Emergency Medicine
FM: Family Medicine
GS: General Surgery
HFO: Health Force Ontario
HiREB: Hamilton Integrated Research Ethics Board
IM: Inpatient Medicine
MD: Medical Doctor (physician)
MOHLTC: Ministry of Health and Long Term Care
OHA: Ontario Hospital Association
OMA: Ontario Medical Association
PA: Physician Assistant
