# Supplementary material for: Understanding health professional role integration in complex adaptive systems: a multiple-case study of physician assistants in Ontario, Canada
Source: BMC Health Serv Res. 2020 Apr 29;20:365. doi: 10.1186/s12913-020-05087-8 (PMC7189743; doi:10.1186/s12913-020-05087-8)
Supplement: Supplementary file 1 — Additional file 1. Sample Interview Guide. [file 12913_2020_5087_MOESM1_ESM.docx]

**Appendix 1: Sample Interview Guide**

**Interview Guide for Supervising Physician**

**A Study to Explore Physician Assistant Integration into Ontario Hospital Settings**

**Interview Orientation**

1. Do you have any questions before we begin?

2. Do I have your permission to start the recording?

**Physician Background and Experience**

3. How did you first hear about physician assistants?

4. How long have you employed or worked with a PA?

a. Can you describe the training/experience your PA brought to their role? (i.e., new grad, military, etc.)

5. Were you the person who made the decision to integrate a PA into your department?

a. If yes, what prompted you to do this? If no, how did you react?

b. Can you describe any resistance or support regarding the PA role in your department/setting?

6. Did you have any previous experience working/employing a PA?

7. How did you feel when you first integrated the PA into your practice?

**Current PA Employer**

8. How many PAs do you employ/supervise in your department?

9. Can you describe to me the role of the PA(s) in your department?

a. What are the PAs responsibilities? What do they do independently and what must they consult you about? How did you come to that understanding?

b. How long did it take for your PA(s) to be efficient in their role?

c. How would you describe the learning curve in working with/integrating a PA?

10. How would you describe the success of the PA role?

11. What are the challenges of employing/supervising a PA in this setting?

12. How would you describe your relationship with the PA? How do you work to improve this relationship? Is the relationship different than with other non-MD health professionals?

13. What are the benefits of working with a PA in a hospital setting/in your department?

14. How do your patients feel about being seen by/with a PA?

15. How do other staff or health care professionals (and who are they) feel about working with a PA?

a. Is your PA part of an interdisciplinary care team?

i. If yes, can you describe the PAs role on the team?

ii. If NP part of team, can you describe difference/similarities between roles?

16. Does your clinic/setting have medical students or residents?

a. If yes, how do they feel about the PA role? What is their understanding?

i. How is the PA introduced or integrated?

ii. How do you distinguish the PA role from the role of a medical resident?

b. What kind of a role does the PA play in terms of mentorship/ teaching/precepting other medical learners or students?

c. If not currently done, do you see a role for the PA to do this?

17. What do you think a PA needs in terms of continuing medical education?

a. What is your role as the supervising physician in maintenance and extension of PA competencies?

b. What are your expectations of the PA in terms of maintenance and extension of PA competencies?

18. What is your understanding about what is happening with PA regulation in the Province?

19. How is your PA funded? Does salary include benefits, CME time?

20. How is liability insurance covered for your PA?

21. In summary:

a. What are the barriers that impact the PA role?

b. What are some examples of where you feel the PA has the biggest impact?

c. What advice would you give a colleague or another department interested in hiring a PA?

22. Are there any other comments or feedback you would like to share? Are there any questions that you feel were missed during this interview that are relevant to the PA role or your experience with a PA?

*End of Interview*
